# A Clinician-Centered Evaluation Framework for Large Language Models in Patient Education: Integrating the Technology Acceptance Model and Medical Condition Regard Scale

**DOI:** 10.2196/100423

**Published:** 2026-09-21

**Authors:** Davis Austria, Grace Lord Williams, Christopher Girardo, Micheal Olaolu Arowolo, Meenakshi Mishra, Charlene Pope, R Neal Axon, Jason Bradley Hill

**Affiliations:** 1 Department of Public Health Sciences, College of Arts and Sciences, Xavier University of Louisiana, 1 Drexel Drive, New Orleans, LA, 70125, United States, 1 917-774-7740; 2 Ochsner Center for Outcomes and Health Services Research, Ochsner Health System, New Orleans, LA, United States; 3 Xavier Ochsner College of Medicine, New Orleans, LA, United States; 4 Department of Pathology, Ochsner Health System, New Orleans, LA, United States; 5 HEROIC, Ralph H Johnson VA Health Care System, Charleston, SC, United States; 6 Hospital Medicine and Innovation, Ochsner Health System, New Orleans, LA, United States

**Keywords:** large language models, patient education, technology acceptance model, Medical Condition Regard Scale, clinical informatics, health informatics, health equity, artificial intelligence, AI, health literacy, readability

## Abstract

Inadequate postcare patient education contributes to preventable readmissions and adverse outcomes that disproportionately affect medically complex, high-need communities. Large language models (LLMs) show promise for generating personalized, plain-language patient education at scale. However, existing LLM evaluation frameworks prioritize technical accuracy over patient accessibility and alignment with health literacy, and few explicitly account for the attitudinal influences that clinician evaluators may introduce into the rating process. In this viewpoint, we introduce an evaluation framework that pairs the Technology Acceptance Model (TAM) with the Medical Condition Regard Scale (MCRS). We call it the TAM-MCRS LLM evaluation framework, a novel clinician-centered approach for comparing which LLMs produce the highest-quality postcare patient education across accuracy, appropriateness, clarity, and completeness. We intend for this framework to be used to evaluate LLM-generated patient education outputs through a 2-arm design that pairs an expert clinician panel with automated assessment methods, allowing for interarm comparison using clinical vignettes while accounting for measured evaluator attitudinal variance. The framework was developed through the National Institutes of Health Artificial Intelligence/Machine Learning Consortium to Advance Health Equity and Researcher Diversity (AIM-AHEAD) Clinicians Leading Ingenuity IN AI Quality (CLINAQ) fellowship program, in partnership with Ochsner Health and Xavier University of Louisiana. The TAM-MCRS framework integrates two theoretical lenses. TAM maps perceived usefulness onto accuracy and completeness, and perceived ease of use onto clarity and appropriateness. Clinicians rate each output with a TAM-based questionnaire, and we then administer the MCRS as a postscoring attitudinal covariate to see whether their regard for the conditions represented in the vignettes influences those ratings. Together, the 2 lenses are intended to produce evidence that is objective, theoretically grounded, clinically realistic, and disparity-responsive. Implications for clinician informaticists, health system governance, and responsible AI deployment are discussed. This viewpoint reflects the authors’ position and is written for clinician informaticists, health system AI governance leaders, implementation scientists, and investigators evaluating LLM-generated patient education.

## Large Language Models in Patient Education: Opportunity and Persistent Gaps

Clinicians, broadly defined in this paper as physicians, advanced practice providers, and nurses involved in patient education and discharge planning, serve as primary translators between medical information and patient understanding. As AI tools increasingly enter clinical environments, health care professionals and clinical informaticists face a pressing question: how do we evaluate whether these tools actually serve our patients, not just in terms of technical accuracy, but also in accessibility, relevance, and responsiveness to diverse patient needs?

The use of large language models (LLMs) in patient-facing applications has grown substantially since the release of publicly accessible generative AI tools. In clinical settings, early use cases have included generating postcare patient education, answering patient portal questions, and producing condition-specific discharge materials. These applications are particularly relevant to clinical practice, where patient education is both a professional standard and a time-intensive responsibility. Inadequate postcare patient education contributes to medication errors, preventable readmissions, and adverse outcomes, with the average cost of a single avoidable readmission estimated at approximately US $15,200 and potentially avoidable emergency department encounters representing approximately US $64.4 billion annually in the United States [1-3].

Research has demonstrated that LLMs can produce patient education content at a level comparable to, and in some cases exceeding, the quality of standard institutional materials [4,5]. However, a critical limitation emerges consistently: most LLM-generated health content defaults to literacy levels that exceed national reading averages [6]. Studies have found that AI-generated patient materials frequently score at a 10th-grade reading level or higher, while health literacy guidelines recommend content at a 6th-grade level for broad accessibility [7]. For patients with limited formal education, older adults, or those navigating health crises, this gap translates directly into reduced comprehension and poorer health outcomes [8].

Equity concerns extend beyond readability. The training data underlying most general-purpose LLMs overrepresent White, English-speaking, and formally educated populations [9]. LLM outputs may therefore reflect cultural assumptions, communication styles, and health beliefs that do not align with the lived experiences of patients from racial and ethnic minority groups, those from lower socioeconomic backgrounds, those with less education, or those navigating language barriers [10]. These barriers reflect what has been described as algorithmic bias by omission, a concept central to the growing literature on health AI [11,12]. These gaps are particularly consequential in regions such as the Louisiana Diabetes Belt and Cancer Alley, where Black, Hispanic, and older adult patients carry disproportionate rates of chronic illness and face compounding barriers to clear health communication [13-17]. In algorithmic development, including these patient demographics alongside conditions and comorbidities is necessary if training corpora are to represent the population they serve.

Our aim in this Viewpoint is to demonstrate how clinician evaluation of LLM-generated patient education should account not only for output quality but also for clinicians’ attitudinal regard toward the medical conditions represented in evaluation scenarios and to propose, as one conceptual approach, a framework pairing the Technology Acceptance Model (TAM) with the Medical Condition Regard Scale (MCRS), which we call the TAM-MCRS LLM evaluation framework. The framework was developed through the National Institutes of Health (NIH) Artificial Intelligence/Machine Learning Consortium to Advance Health Equity and Researcher Diversity (AIM-AHEAD) Clinicians Leading Ingenuity IN AI Quality (CLINAQ) fellowship program at Xavier University of Louisiana in partnership with Ochsner Health, with foundational conceptualization supported through the Veterans Affairs Quality Scholars fellowship program. Within the fellowship, the framework was designed to evaluate the performance of LLMs in generating high-quality postcare patient education across accuracy, appropriateness, clarity, and completeness, using clinical vignettes and evaluation by an expert clinician panel. We write for clinician informaticists, health system AI governance leaders, implementation scientists, and investigators evaluating LLM-generated patient education.

The remainder of this Viewpoint proceeds in 3 moves. We first set out the 2 lenses the framework rests on, explaining what TAM contributes to judging whether patient education is usable and what the MCRS contributes to judging the clinicians’ attitudinal variation. We then describe why the two belong together and what neither achieves alone. Finally, we present the framework itself, a 2-arm design in which clinician and automated evaluation are applied to the same outputs and compared, and we close with what this means for practice, health system governance, and the discipline.

## The Foundation of Our Framework: Benefits and Limitations

### The TAM: A Theoretically Grounded Lens for Technology Adoption

TAM, originally proposed by Davis in 1989 [18] and subsequently extended by Venkatesh et al [19], has become one of the most frequently applied frameworks in health informatics. Historically used to evaluate the adoption of electronic health records, patient portals, telehealth platforms, and clinical decision support systems, this scope of use has since expanded to evaluations of AI tools in clinical settings [19,20]. While TAM’s constructs of perceived usefulness (PU) and perceived ease of use (PEOU) provide a solid foundation for understanding technology adoption, TAM posits that PU and PEOU determine only the behavioral intention to adopt a technology. Therefore, their application to LLM evaluation in patient education has remained incomplete for a clinically important reason: TAM alone cannot account for how clinicians’ attitudes toward a patient’s medical condition shape their judgment of an LLM output’s quality, relevance, and appropriateness.

Applied to LLM evaluation in patient education, TAM’s constructs map productively onto the evaluation problem. PU corresponds to whether an LLM output contains accurate, complete, and guideline-adherent information that a clinician would trust. PEOU corresponds to whether the output is written in plain language that patients with varying health literacy levels can readily understand. These mappings are clinically meaningful (Table 1). However, they leave a critical evaluative gap unaddressed: when expert clinicians serve as proxy raters of patient-facing AI outputs, their judgments are filtered through attitudes, assumptions, and levels of regard toward the clinical conditions and patient populations being evaluated.

**Table 1.** Mapping of Technology Acceptance Model (TAM) constructs to the 4 TAM-Medical Condition Regard Scale (MCRS) evaluation criteria.

TAM construct	Definition	Evaluation criterion	Guiding evaluation question
Perceived usefulness	The degree to which a person believes using a technology would enhance their performance	Accuracy	Does the output contain guideline-adherent, actionable information a clinician would trust?
Perceived usefulness	The degree to which a person believes using a technology would enhance their performance	Completeness	Does the output cover all required clinical and social determinants of health (SDoH) elements a patient needs to act safely?
Perceived ease of use	The degree to which a person believes using a technology would be free of effort	Clarity	Is the output written in plain language at or below a 6th-grade reading level per Agency for Healthcare Research and Quality standards?
Perceived ease of use	The degree to which a person believes using a technology would be free of effort	Appropriateness	Does the output reflect the patient’s specific SDoH context, language preference, and communication needs?

### The MCRS: A Theoretically Grounded Lens for Attitudinal Variance

The MCRS, developed by Christison et al [21], is a validated instrument that measures clinicians’ attitudes and regard toward patients with specific medical diagnoses. Originally applied in medical education to assess attitudinal confounds toward stigmatized conditions such as substance use disorders, obesity, and mental illness, the MCRS captures a dimension of clinical evaluation that technical rubrics systematically miss—the attitudinal context in which clinician judgment operates.

In the context of LLM evaluation for patient education, MCRS-inspired constructs address a specific and underexamined validity threat. When an expert clinician panel rates LLM-generated postcare patient education for a patient with depression, opioid use disorder, or morbid obesity, their attitudinal regard for that condition may influence how they score the output’s appropriateness, tone, and personalization, independent of the LLM’s actual performance. Without accounting for this dimension, clinician panel ratings may reflect evaluator attitudinal variance as much as model quality, undermining the validity of the evaluation.

## TAM-MCRS: A Combination Prescription

The TAM-MCRS LLM evaluation framework addresses a gap that existing evaluation approaches were not designed to close. Most published clinical LLM evaluations measure performance against clinician standards, asking whether a model’s output is accurate enough for a clinician to trust. This framework reorients the evaluative question in two directions simultaneously: is the output accessible, personalized, and equitable enough for a patient to use, and how might measured clinician attitudinal variance influence the ratings used to answer that question?

The first reorientation reflects a core health informatics principle: technology adoption is not determined by technical performance alone, but by the PU and PEOU experienced by the people the technology is meant to serve [18]. The second reorientation, introducing the MCRS as an attitudinal covariate, reflects the discipline’s accountability to equity. Clinician panels are the gold standard for LLM evaluation in health care, but they are not attitudinally neutral. A framework that does not account for this difference is not fully valid for disparity-responsive research.

The stratified, disparity-responsive analysis built into this framework, which examines model performance across vignette race, language, social determinants of health (SDoH) tier, and age, positions equity not as an add-on but as a core dimension of evaluation. Prior research has documented that LLMs are less accurate for populations underrepresented in their training data [22] and less factually precise for subjects that appear there infrequently [23]. This framework applies that insight to patient demographics, testing whether performance gaps emerge systematically for underserved patient profiles. The MCRS covariate analysis adds a second disparity-responsive validity layer, ensuring that observed performance gaps reflect model differences rather than evaluator attitudinal variance.

Multiple frameworks for evaluating LLMs in health care have emerged in recent years, including comprehensive human-evaluation frameworks such as QUEST (Quality of Information, Understanding and Reasoning, Expression Style and Persona, Safety and Harm, and Trust and Confidence), which organizes clinician evaluation around quality of information, understanding and reasoning, expression style and persona, safety and harm, and trust and confidence [24], and multidimensional benchmarking frameworks such as MEDIC (medical reasoning, ethics and bias, data and language understanding, in-context learning, and clinical safety), which assesses medical reasoning, ethics and bias, data and language understanding, in-context learning, and clinical safety [25]. Recent surveys of LLM evaluation in health care similarly observe that prior evaluation has focused narrowly on accuracy and call for broader, multidimensional criteria spanning safety, reasoning, and clinical applicability [26]. Separately, TAM is widely used to study clinician adoption of health technologies [20], and the MCRS measures clinician attitudes toward specific patient conditions [21]. To our knowledge, however, no existing framework integrates these 2 instruments as paired, measured constructs within a clinician-centered evaluation of LLM-generated patient education. Existing work occupies adjacent space, applying TAM to technology adoption or proposing standardized human-evaluation workflows for health care LLMs. However, none combines TAM and MCRS to model clinician usability perceptions and condition-specific regard as analytic covariates alongside output quality, readability, and understandability.

## Our TAM-MCRS LLM Evaluation Framework

### Conceptual Architecture

**Figure 1.**
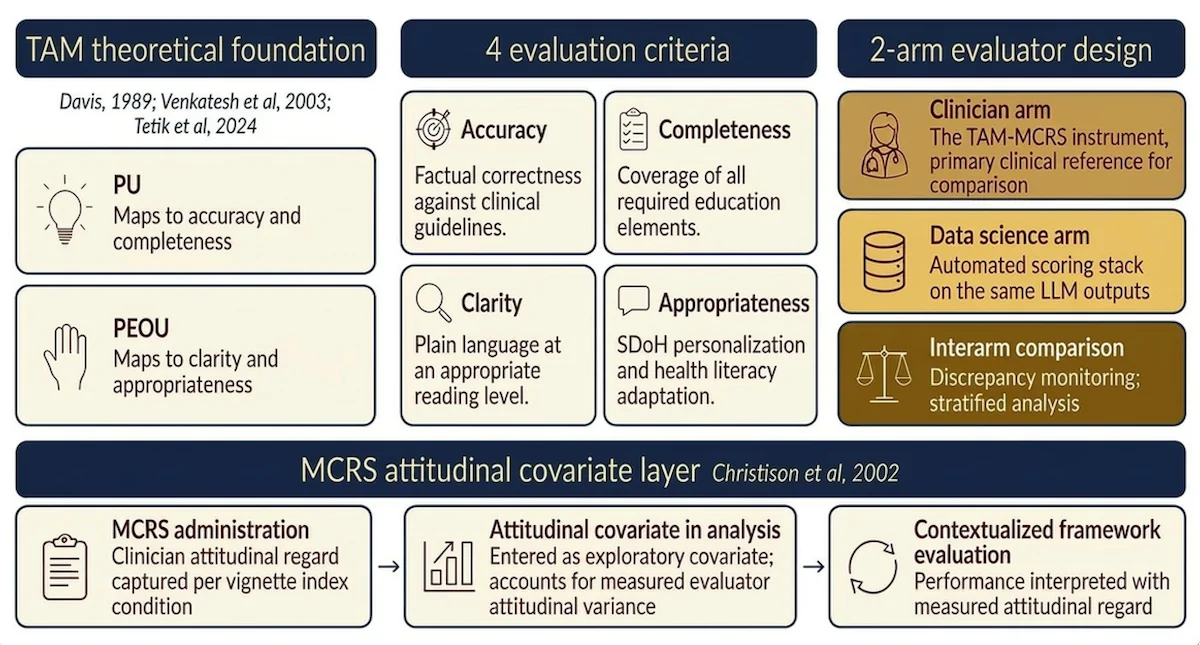
Technology Acceptance Model-Medical Condition Regard Scale (TAM-MCRS) conceptual framework for clinician-centered large language model (LLM) evaluation in patient education. TAM constructs (perceived usefulness [PU] and perceived ease of use [PEOU]) map onto 4 evaluation criteria [18-20]. The MCRS serves as the attitudinal covariate layer, supporting examination of measured evaluator attitudinal variance in clinician panel ratings [21]. SDoH: social determinants of health.

We organized our framework around a 2-arm evaluator design that can be applied to the outputs of one or more models. In the clinician arm, an expert clinician panel evaluates each output with the TAM-MCRS instrument, which integrates 4-criterion quantitative scoring with the MCRS attitudinal regard measure, serving as the primary clinical reference for comparison. In the data science arm, automated methods score the same outputs independently. Automated scores are used for parallel assessment and discrepancy monitoring. When automated scores diverge from clinician ratings, the divergence should be reported descriptively rather than resolved through adjudication. The MCRS attitudinal covariate contextualizes the clinician ratings. Together, these components produce a replicable, multidimensional approach to assessing LLM-generated patient education that accounts for both output quality and the attitudinal context in which clinician evaluation operates (Figure 1).

**Figure 2.**
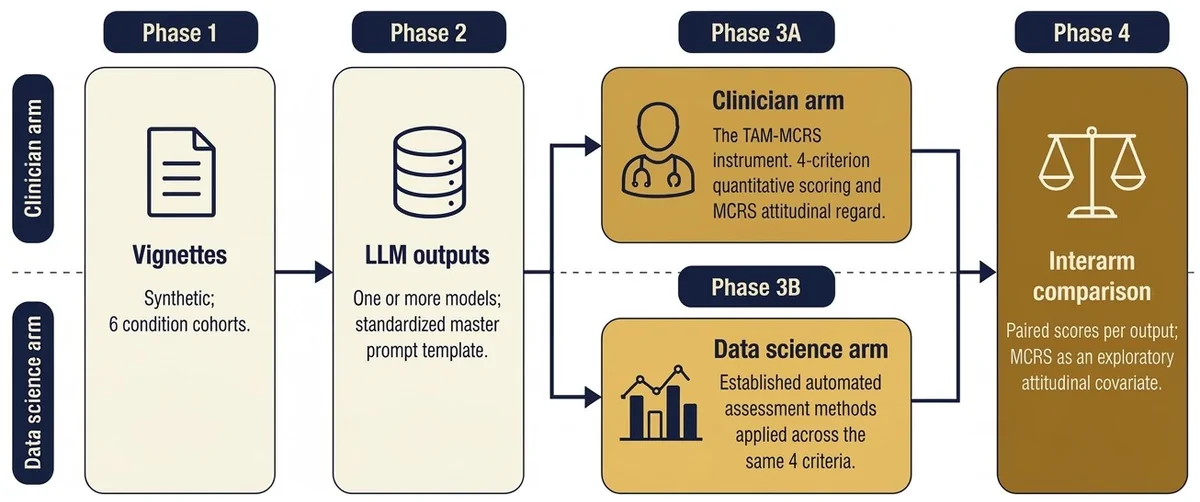
Proposed Technology Acceptance Model-Medical Condition Regard Scale (TAM-MCRS) large language model (LLM) evaluation workflow. The framework begins with clinically realistic synthetic vignettes, generates patient education outputs from one or more models using a standardized master prompt template, and evaluates these outputs across 2 arms. The clinician arm administers the TAM-MCRS instrument, which combines 4-criterion quantitative scoring with MCRS attitudinal regard items. The data science arm applies established automated assessment methods across the same 4 criteria. Paired scores are then compared across arms, with the MCRS score used as an exploratory covariate.

The MCRS-inspired survey layer contextualizes the quantitative ratings by surfacing attitudinal influences that may have shaped clinician judgment, providing both a validity check and a source of qualitative data for framework refinement (Figure 2).

### The Clinician Arm

In the clinician arm, an expert panel works through a single structured TAM-MCRS instrument. The TAM component captures each output’s PU and PEOU from a patient perspective; because these constructs operationalize the questions this framework asks, they serve as criterion dimensions rather than adoption predictors. The MCRS component is adapted as a postevaluation covariate, retaining the core domains of clinician regard. Clinicians rate their regard toward the diagnosis represented in the vignette, not toward the output itself, so that attitudinal regard can be examined as a possible source of rating variance independent of the model that produced it [21]. Because the adapted instrument has not been independently validated in this configuration, its use is explicitly exploratory. The criterion mapping shown in Table 1 governs which instrument items inform which score.

### The Data Science Arm

In the data science arm, outputs from each model are evaluated against the 4 primary TAM criteria, with each criterion matched to an evidence-based scoring method (Table 2). The 4 criteria and their scoring approaches were designed to address the specific limitations of existing LLM benchmarks, which rely primarily on n-gram statistical approaches such as BLEU (Bilingual Evaluation Understudy) [27] and ROUGE (Recall-Oriented Understudy for Gisting Evaluation) [28]; these approaches cannot capture semantic nuance, population-specific communication needs, or health literacy adaptation.

**Table 2.** Four-criterion Technology Acceptance Model-Medical Condition Regard Scale (TAM-MCRS) evaluation matrix.

Criterion	Definition
Accuracy	Factual correctness of diagnoses, medications, red flags, and follow-up vs clinical guidelines
Appropriateness	Social determinants of health personalization, health literacy adaptation, and population-specific communication needs
Clarity	Plain language quality, reading level, and patient-facing tone
Completeness	Coverage of all required education elements per vignette condition

Table 2 defines each criterion. Two features matter for the argument rather than the mechanics. Clarity carries an a priori directional hypothesis: a biomedical training corpus may elevate clinical vocabulary at the expense of accessibility, so a model that scores well on accuracy may score poorly on the dimension that decides whether a patient can act. Completeness is scored in both arms, which is what makes the interarm comparison possible. The content checklist is provided in Multimedia Appendix 1 and an illustrative vignette in Multimedia Appendix 2. In future empirical applications, established automated methods such as FActScore [23], G-Eval [29], and Flesch-Kincaid readability with the Patient Education Materials Assessment Tool (PEMAT) [30] may support these criteria alongside clinician judgment.

To make this concrete, consider how the framework would operate in practice. A health system is choosing between 2 language models to draft discharge instructions. The patient is an older adult with hypertension and type 2 diabetes, on multiple medications, facing transportation and cost barriers to filling prescriptions. Both models generate the patient’s instructions from the same prompt. A clinician on the panel reads each version and scores it on the 4 criteria, without seeing which model produced it, then records their regard for the conditions in the vignette. Meanwhile, the automated pipeline scores the same 2 outputs on the same 4 criteria. The system now has 2 independent readings of each output, a measure with which to examine whether the rater’s own attitude may have shaped their scores, and a defensible basis for choosing between the models.

## Our Future Directions

This framework is designed to serve as the evaluative foundation for a subsequent Readability, Disparity-responsive design, and Personalization prompting framework and a future multisite R01 clinical trial examining AI-enabled postcare communication quality across safety-net health systems. When applying our framework in evaluations, criterion scores would be compared across model groups, with the MCRS score entered as an exploratory covariate to assess whether condition-specific clinician regard is associated with rating variation. Repeated ratings and the measurement properties of each criterion would be addressed using appropriate statistical methods, as detailed in the subsequent empirical report. Exploratory stratification would examine whether performance differences vary by vignette race or ethnicity, language preference, SDoH tier, and age group. Future applications of the framework, whether by our collective or others, should publicly document model versions, model prompts, scoring prompts, rubric specifications, and analytic assumptions before data collection to support reproducibility. Full analytic specification, including model syntax and prespecified hypotheses, is provided in the accompanying materials. We recognize that the primary limitation of the framework is that the PEOU scores are currently designed to be provided by clinician proxies rather than by patients themselves. In the future, we are dedicated to incorporating direct patient evaluators from the target populations, including those in the Louisiana Diabetes Belt and Cancer Alley, to cross-validate clinician proxy assessments of patient-facing accessibility against the health literacy and communication barriers experienced by the populations this framework is designed to serve.

## From Bedside to Boardrooms: Framework Impact

At the bedside, informaticists and clinical educators can use the 4-criterion rubric as a practical vetting tool before deploying any LLM-generated patient education material. The checklist-based completeness scoring and Flesch-Kincaid grade-level targets are straightforward to apply without specialized AI expertise, making the framework accessible to frontline staff in postcare communication workflows.

At the health system level, the framework provides a structured basis for LLM procurement and governance decisions. Health systems currently adopt LLMs with limited standardized evaluation criteria for patient-facing applications. A reproducible, multimethod evaluation framework gives informatics leaders a defensible, evidence-based process for comparing tools before deployment, with a documentation trail that supports institutional review board and regulatory accountability.

At the disciplinary level, this framework advances health informatics as a field that evaluates not only whether AI tools work but also whether they work responsibly and whether our methods for determining that are themselves free from attitudinal confounders. As LLMs become increasingly embedded in clinical workflows, informaticists are uniquely positioned to lead evaluation efforts that center patient experience, clinician accountability, and population health outcomes.

## Supplementary material

10.2196/100423 Multimedia Appendix 1 Proposed master prompt template. The standardized prompt template we propose is to be applied uniformly across all models evaluated at temperature 0. It includes a Flesch-Kincaid grade-level target, 7-element content checklist, social determinants of health context injection fields, patient-centered framing directives, section headers for completeness scoring, dynamic length guidance for reusable patient education workflows, and mandatory safety element requirements. For the Clinicians Leading Ingenuity IN AI Quality (CLINAQ) application in which we propose to use it, the target is the multimorbidity and polypharmacy pathway at 500 to 650 words.

10.2196/100423 Multimedia Appendix 2 Illustrative sample clinical vignette. A representative synthetic, deidentified clinical vignette (hypertension with type 2 diabetes comorbidity) is provided to illustrate the proposed evaluation workflow, reflecting the multimorbidity, polypharmacy, and social determinants of health (SDoH) complexity the framework is designed to address. It includes patient profile, medication list, SDoH fields, and evaluation scoring guidance.

## Abbreviations

AIM-AHEAD: Artificial Intelligence/Machine Learning Consortium to Advance Health Equity and Researcher Diversity
BLEU: Bilingual Evaluation Understudy
CLINAQ: Clinicians Leading Ingenuity IN AI Quality
LLM: large language model
MCRS: Medical Condition Regard Scale
MEDIC: medical reasoning, ethics and bias, data and language understanding, in-context learning, and clinical safety
PEMAT: Patient Education Materials Assessment Tool
PEOU: perceived ease of use
PU: perceived usefulness
QUEST: Quality of Information, Understanding and Reasoning, Expression Style and Persona, Safety and Harm, and Trust and Confidence
ROUGE: Recall-Oriented Understudy for Gisting Evaluation
SDoH: social determinants of health
TAM: Technology Acceptance Model
