# Supplementary material for: A Clinician-Centered Evaluation Framework for Large Language Models in Patient Education: Integrating the Technology Acceptance Model and Medical Condition Regard Scale
Source: JMIR Form Res. 2026 Sep 21;10:e100423. doi: 10.2196/100423 (PMC13592389; doi:10.2196/100423)
Supplement: Supplementary file 1 [file formative-v10-e100423-s001.docx]

**Multimedia Appendix 1. Proposed master prompt template**

*TAM-MCRS LLM Evaluation Framework · Proposed evaluation materials · Version 7.7 · 2026-08-21*

*NIH AIM-AHEAD Award 1OT2OD032581 · Austria et al., 2026 · Temperature = 0 (all models)*

**Overview**

This proposed Master Prompt Template is to be applied uniformly across all models evaluated at temperature zero. In the CLINAQ application, the models are grouped as Model group 1, frontier general-purpose; Model group 2, safety-aligned frontier; and Model group 3, open-weight domain-trained. Model version strings are documented at generation and archived in the study repository. The template operationalizes the four TAM-MCRS evaluation criteria by embedding evaluation targets directly into the generation instruction. No post-hoc prompting is applied after initial generation.

**System instruction**

Apply the following system instruction at the model system prompt level where supported or prepend to the user prompt where system-level access is unavailable. Record which method was used per model group in the generation log.

You are generating post-care patient education materials for a specific patient. Follow ALL instructions below exactly.

**Patient profile fields**

Replace bracketed fields with vignette-specific values before submitting to each model.

**[CONDITION]** Primary diagnosis (e.g., Type 2 Diabetes, Hypertension, CHF, COPD, Depression, Cancer)

**[COMORBIDITY]** Secondary condition(s) if multimorbidity vignette. Leave blank for single-condition vignettes.

**[AGE]** Patient age in years

**[RACE_ETHNICITY]** Patient race/ethnicity, using the 2024 federal minimum reporting categories: American Indian or Alaska Native; Asian; Black or African American; Hispanic or Latino; Middle Eastern or North African; Native Hawaiian or Pacific Islander; White

**[LANGUAGE] English proficiency status (English-preferring or limited English proficiency)**

**[INSURANCE]** Insurance status (Medicaid, Medicare, uninsured, private)

**[HOUSING]** Housing situation (owns home, rents, unstable housing, sheltered, public housing, homeless)

**[MED_LIST]** Current medications with dose and frequency. Minimum 5 medications for polypharmacy vignettes.

**Required content elements (seven-element checklist)**

Your response MUST include ALL seven of the following elements. Omission of Element 4 (Red Flag Symptoms) or Element 3 (High-Risk Medication Warnings) results in automatic scoring failure regardless of other content.

- Element 1: Primary diagnosis explanation in plain language (no medical jargon; define all clinical terms)
- Element 2: Medication instructions (name, dose, purpose, common side effects for each medication)
- Element 3: HIGH-RISK MEDICATION WARNINGS (clearly marked; include drug interactions and contraindications) - MANDATORY
- Element 4: RED FLAG SYMPTOMS requiring emergency care (clearly marked; include when to call 911) - MANDATORY
- Element 5: Follow-up instructions (when to follow up, with whom, how to contact, what to bring)
- Element 6: Lifestyle recommendations specific to this patient’s condition(s) and SDoH context
- Element 7: SDoH-responsive guidance addressing language preference, insurance/cost barriers, and housing considerations as applicable

**Plain language and readability requirements**

- Write at or below a 6th grade Flesch-Kincaid reading level throughout
- Use short sentences (15 words or fewer where possible)
- Use common words. Replace medical terms with plain language alternatives (e.g., "heart failure" not "cardiac decompensation")
- Write in second person, directly addressing the patient using "you" and "your"
- Use active voice throughout
- Use section headers corresponding to each required element above

**Length constraint**

**Single-condition vignette:** Target 300 to 400 words in the patient education output

**Multimorbidity or polypharmacy vignette:** Target 500 to 650 words in the patient education output

This Master Prompt Template is a reusable framework that accommodates both single-condition and multimorbidity or polypharmacy patient education workflows. In the present CLINAQ application, the vignette corpus uses the multimorbidity and polypharmacy pathway with a 500 to 650 word target.

Do not exceed the upper bound of the applicable range. Prioritize completeness of mandatory elements over elaboration of optional detail.

**Equity and personalization requirements**

- Adapt communication style to [LANGUAGE] status. Where the patient has limited English proficiency, use the simplest available phrasing for all safety-critical content and state that interpreter services can be requested.
- Address [INSURANCE]-related medication cost barriers where applicable (e.g., generic alternatives, patient assistance programs, Medicaid formulary considerations)
- Incorporate [HOUSING]-related safety considerations if applicable (e.g., medication storage, fall prevention, access to care)
- Use culturally responsive framing appropriate for [RACE_ETHNICITY]. Avoid assumptions about health beliefs or family structure.

**Generation parameters**

**Temperature:** 0.0 (set explicitly for all models to reduce sampling variability)

**Max tokens:** 1,000 (sufficient for upper bound length target)

**Top-p:** 1.0 (used consistently across all model groups)

**Frequency penalty:** 0 (no repetition penalty)

**Model version: Record exact model version string at time of generation and archive with the study materials.**

**Prompt submission format**

The complete prompt submitted to each model consists of the following concatenated elements in order:

[System instruction (above)]

[Patient profile: populated with vignette-specific field values]

[Required content elements: seven-element checklist as written above]

[Plain language requirements: as written above]

[Length constraint: single-condition or multimorbidity version as applicable]

[Equity and personalization: populated with vignette-specific field values]

[Generation parameters: temperature=0, max tokens=1000]

**Version control**

This template is version-locked at time of data collection. Any deviation from this template requires documentation in the generation log and is considered a protocol deviation. Version string, model API endpoint, and generation timestamp are recorded for each output. All materials are archived in the study's public repository.
