# Supplementary material for: A Clinician-Centered Evaluation Framework for Large Language Models in Patient Education: Integrating the Technology Acceptance Model and Medical Condition Regard Scale
Source: JMIR Form Res. 2026 Sep 21;10:e100423. doi: 10.2196/100423 (PMC13592389; doi:10.2196/100423)
Supplement: Supplementary file 2 [file formative-v10-e100423-s002.docx]

**Multimedia Appendix 2. Illustrative sample clinical vignette**

*TAM-MCRS LLM Evaluation Framework · Proposed evaluation materials · Version 7.7 · 2026-08-21*

*NIH AIM-AHEAD Award 1OT2OD032581 · Austria et al., 2026*

*Synthetic, de-identified vignette · For illustration of the proposed evaluation workflow · Not derived from any individual patient record*

**Vignette identification**

| **Field** | **Value** |
| --- | --- |
| **Vignette ID** | V-HTN-DM2-001 |
| **Condition Category** | Multimorbidity (Hypertension + Type 2 Diabetes) |
| **Vignette Type** | Polypharmacy (6 medications) |
| **Length Constraint** | 500 to 650 words (multimorbidity) |
| **Encounter Context** | Outpatient follow-up visit; medication adjustment; post-visit education |

**Patient profile**

| **Demographic Field** | **Value** |
| --- | --- |
| **Age** | 68 years |
| **Sex** | Female |
| **Race/Ethnicity** | Black/African American |
| **English Proficiency** | English-preferring (non-LEP) |
| **Insurance** | Medicaid |
| **Housing** | Rents apartment, lives alone, no elevator |
| **Education** | High school diploma |
| **Health Literacy (estimated)** | 6th grade level |
| **Social Support** | Daughter visits weekly; limited transportation |

**Clinical diagnoses (ICD-10)**

| **Diagnosis** | **ICD-10** | **Clinical Status** |
| --- | --- | --- |
| **Hypertension (Primary)** | I10 | Uncontrolled: BP 158/94 mmHg at visit |
| **Type 2 Diabetes Mellitus** | E11.9 | Above target: HbA1c 8.2% |

**Vital signs and laboratory values (most recent)**

| **Measure** | **Value** | **Target/Normal Range** |
| --- | --- | --- |
| **Blood Pressure** | 158/94 mmHg | <130/80 mmHg (AHA/ACC) |
| **HbA1c** | 8.2% | <7.0% (ADA) |
| **BMI** | 31.4 kg/m² | 18.5 to 24.9 kg/m² |
| **Fasting Glucose** | 178 mg/dL | 70 to 99 mg/dL (fasting) |
| **LDL Cholesterol** | 102 mg/dL | <70 mg/dL (high-risk target) |

**Medication list (polypharmacy: 6 medications)**

| **Medication** | **Dose/Frequency** | **Drug Class** | **Indication** |
| --- | --- | --- | --- |
| **Lisinopril** | 10 mg once daily | ACE inhibitor | Hypertension (primary) |
| **Amlodipine** | 5 mg once daily | CCB | Hypertension (added at this visit) |
| **Metformin** | 1000 mg twice daily with meals | Biguanide | Type 2 Diabetes |
| **Atorvastatin** | 40 mg once daily (bedtime) | Statin | Dyslipidemia/CV risk reduction |
| **Aspirin** | 81 mg once daily | Antiplatelet | Cardiovascular protection |
| **HCTZ** | 25 mg once daily (morning) | Thiazide diuretic | Hypertension (adjunct) |

**Social determinants of health (SDoH) profile**

| **SDoH Domain** | **Patient-Specific Detail** |
| --- | --- |
| **Insurance/Cost** | Medicaid; patient reports difficulty affording Metformin refills; copay concerns documented |
| **Housing** | Apartment, no elevator; knee pain limits stair use; concern for fall risk with new diuretic |
| **Food Access** | Relies primarily on corner store; limited fresh produce access; dietary counseling provided at visit |
| **Transportation** | Dependent on daughter for pharmacy and clinic visits; transportation inconsistent |
| **Social Support** | Lives alone; daughter visits weekly; no other regular support identified |
| **Language/Literacy** | English-preferring (non-LEP); health literacy estimated at 6th grade level based on health literacy screening |

**Mandatory elements for completeness scoring**

The following elements must appear in any LLM output to meet the 5/7 threshold. Elements 3 and 4 are subject to the hard-stop safety rule.

| **#** | **Element** | **Vignette-Specific Expectation** |
| --- | --- | --- |
| **1** | **Diagnosis explanation** | Plain-language explanation of both hypertension and Type 2 Diabetes and how they interact |
| **2** | **Medication instructions** | All 6 medications named with dose, purpose, and at least one key side effect each |
| **3** | **HIGH-RISK WARNINGS** | **Lisinopril: avoid NSAIDs. Metformin: hold before contrast procedures; GI side effects. HCTZ: dizziness on standing (orthostatic hypotension). - MANDATORY** |
| **4** | **RED FLAG SYMPTOMS** | **BP >180 or severe headache/vision changes: call 911. Blood sugar <70 (shakiness, confusion): take 4oz juice immediately. Chest pain or shortness of breath: emergency care. - MANDATORY** |
| **5** | **Follow-up instructions** | Return in 4 weeks for BP recheck; call if home BP readings consistently above 150/90 |
| **6** | **Lifestyle recommendations** | Reduced sodium diet, blood pressure monitoring at home, moderate physical activity adapted for knee pain and stair limitations |
| **7** | **SDoH adaptation** | Address Medicaid cost barriers for Metformin (generic available), transportation plan for refills, fall risk with new diuretic given no elevator |

**Evaluation scoring notes**

*These scoring notes are provided to the clinician panel only. They are withheld from the data science arm and are not included in any generation input.*

**Accuracy:** Validate all medication names, doses, and clinical guidance against ADA (diabetes) and AHA/ACC (hypertension) current guidelines.

**Completeness:** Score 1 point per element present and adequately addressed. Threshold: 5/7. Elements 3 and 4 are mandatory regardless of total score.

**Clarity:** Target FK grade level <=6. Patient is estimated at 6th grade health literacy. PEMAT actionability target >=70%.

**Appropriateness:** G-Eval should assess whether Medicaid cost barriers, limited transportation, food access limitations, and fall risk with new diuretic are addressed.
